# Supplementary material for: Toward neuroanatomical and cognitive foundations of macaque social tolerance grades
Source: eLife. 2026 Mar 3;14:RP106424. doi: 10.7554/eLife.106424 (PMC12956280; doi:10.7554/eLife.106424)
Supplement: Supplementary file 1. [file elife-106424-supp1.docx]

|  | CdP | Utrecht University | Inserm  Oxford University | Japan Monkey Center | Total |
| --- | --- | --- | --- | --- | --- |
| *M. arctoides [3]* |  | 3 |  |  | 3 |
| *M. fascicularis [2]* | 3 | 1 | 1 | 1 | 6 |
| *M. fuscata [1]* |  | 2 |  | 2 | 4 |
| *M. mulatta [1]* | 8 | 1 | 4 |  | 13 |
| *M. nemestrina [2]* | 1 | 1 |  |  | 2 |
| *M. nigra [4]* | 1 | 1 |  |  | 2 |
| *M. silenus [3]* |  | 1 |  |  | 1 |
| *M. sinica [3]* |  |  |  | 1 | 1 |
| *M. sylvanus [3]* |  | 2 |  |  | 2 |
| *M. thibetana [2]* | 1 |  |  |  | 1 |
| *M. tonkeana [4]* | 6 |  |  |  | 6 |
| *M. radiata [3]* |  |  |  | 1 | 1 |
| Total | 20 | 12 | 5 | 5 | 42 |

**Supplementary file S1: Species and data collection centers in the dataset**. Summary table of our dataset detailing the macaque species, the social grades [x] and the centers they originate from. The dataset includes brain imaging data from 42 individuals across 12 macaque species. Columns represent the collection centers: CdP (Centre de Primatologie de l’Université de Strasbourg, France), Utrecht University (Netherlands), INSERM-Oxford University (Fr/UK), and Japan Monkey Center (Japan). Each row corresponds to a macaque species, with the number of individuals collected from each center. The total count per species and per center is indicated in the final column and row, respectively. The social grade of the species is indicated between brackets.
