# Supplementary material for: Toward neuroanatomical and cognitive foundations of macaque social tolerance grades
Source: eLife. 2026 Mar 3;14:RP106424. doi: 10.7554/eLife.106424 (PMC12956280; doi:10.7554/eLife.106424)
Supplement: Supplementary file 2. [file elife-106424-supp2.docx]

| **Materials** | **Description** |
| --- | --- |
| Multifunctional rotary tool (Dremel type) associated with a sawing disc | To precisely cut out and remove the skull and have access to the brain. |
| Osteotome | To separate the skull from the atlas. |
| Mallet | Used with the osteotome. |
| Hammer-axe | To separate the skull from the atlas and to enlarge the space between the muscular planes and the skull. |
| Autopsy knife | To cut out the flesh and muscles. |
| Scalpel handle and blade | To dissect the scalp, muscles of the head, the dura, and the facial nerves. |
| Grooved probe | To dissect the dura without damaging the underlying tissues. |
| Pair of curved scissors with round tip | To cut out flesh at various steps of the dissection. |
| Dissecting forceps with and without claws | To hold tissues without damaging them to perform sections (skin, muscles, dura, nerves). |
| Graded beakers | To measure and transfer formaldehyde into the bucket for the brain fixation. |
| Funnel | To transfer the various liquids without spilling. |
| Test tube with graduation | To measure out the required amount of distilled water for the PBS. |
| Buckets (x4) | Containers for the brain fixation. |
| Hemispheric receptacles | With holes at regular intervals, to hold the brain spherical shape whilst it is immersed in formaldehyde. |
| Plastic jar with screw lid | Transparent, 7 centimeters in diameter (adapted to the size of the MRI antenna used). Container for brain during MRI acquisitions. |
| Fluorinert^TM^ FC-770 | Fluorocarbon-based fluid, helps to displace air bubbles^[1]^ and improves brain/background image delineation. |
| Distilled water | To dissolve the PBS tabs to make PBS solution. |
| Parafilm | To seal the MRI containers and prevent any leakage. |
| Cotton batting/aquarium foam squares | To place inside the MRI container at the bottom, the top and on one side. Used to pad the brain specimen and contain the bubble at the top of the container. |
| FFP2 mask | Safety measures for zoonosis. |
| Latex or nitril surgical gloves | For handling specimen brains. |
| Lab coats (or surgical gowns) | For individual protection whilst performing the dissection. |
| Marker | To label the various containers (mentioning if it has contained formaldehyde), label the brain specimen. |
| 7T MRI Antenna | For the MRI acquisition (whole brain acquisition). Check the internal diameter to choose the adequate container. |
| **Material for the use of paraformaldehyde** | |
| Formaldehyde solution 4 % | pH 6,9 solution for histological tissue fixation. Ordered from Sigma-Aldrich (Product ID: HT501640-19L).]  At least 10 x volume of a macaque brain (roughly 3 liters per brain). |
| Formaldehyde spill response kits | Used for safety in case of any formaldehyde spilling. |
| Fume hood | To manipulate formaldehyde ensuring the safety of the experimenter. |
| Face shield | For the experimenter’s safety in case of any spilling. |
| Mask | For the experimenter’s safety in case of any spilling. |
| Gloves (chemical resistant) | For the experimenter’s safety for handling the formaldehyde. |
| Baritainer | For discarding waste |

^[1]^ ^5^

**Supplementary file S2: Necessary materials for NHP brain extraction.** Summary table of the tools required for the brain extraction we performed, including their names and descriptions.
