# Supplementary material for: Toward neuroanatomical and cognitive foundations of macaque social tolerance grades
Source: eLife. 2026 Mar 3;14:RP106424. doi: 10.7554/eLife.106424 (PMC12956280; doi:10.7554/eLife.106424)
Supplement: Supplementary file 3. [file elife-106424-supp3.docx]

| MRI Center | Primatology Center  (with ICube laboratory) | Utrecht University | Japan Monkey Center | Oxford University |
| --- | --- | --- | --- | --- |
| Number of individuals | 20 | 14 | 4 | 4 |
| MRI | Bruker Biospec 7T 70/30 UR | Agilent Variant 9.4T | Bruker Biospec 9.4T 90/20 UR | Clinical Horizontal 3T |
| Antenna | Volume coil | Surface coil | Volume coil | Volume coil |
| MRI weighting | T2  Sequence: Turbo_RARE_3D | T1 | T2 | T2 |
| Spatial resolution | 0.20 x 0.20 x 0.20 mm | 0.25 x 0.25 x 0.25 mm | 0.25 x 0.25 x 0.25 mm | 0.6 x 0.6 x 0.6 mm |
| Brain fixation characteristics | Immersion: Formaldehyde 4% | Perfusion: Formaldehyde 4% | Immersion: formaline 10% | Perfusion: Formaldehyde 4% |
| Fluid used for scanning | Fluorinert^®^ FC-770 | Sucrose 15%, 4°C | 3M^TM^ Fluorinert | Fomblin or Fluorinert^®^ FC-770 |

**Supplementary file S3: Information relating to the acquisition of anatomical MRI images and the procedures for fixing and preserving post-mortem samples according to the different centers.** Summary table of the MRI acquisitions parameters and *post-mortem* sample preservation protocols across centers. The final dataset consists of 42 anatomical scans after pruning data with missing age or sex information (10 individuals), with both T1 and T2-weighted images. Due to their different origins, the images in the dataset did not follow the exact same acquisition protocols (different scanners and acquisition parameters). In addition, post-mortem brain preservation and perfusion protocols are different, which may also influence the images obtained.
